# Supplementary material for: Ubiquitin-derived artificial binding proteins targeting oncofetal fibronectin reveal scaffold plasticity by β-strand slippage
Source: Commun Biol. 2024 Jul 27;7:907. doi: 10.1038/s42003-024-06569-9 (PMC11283464; doi:10.1038/s42003-024-06569-9)
Supplement: Supplementary file 2 — Supplementary Information [file 42003_2024_6569_MOESM2_ESM.pdf]

Supplementary Information

"Ubiquitin-derived artificial binding proteins targeting oncofetal fibronectin reveal scaffold plasticity by β-strand slippage"

Anja Katzschmann, Ulrich Haupts, Anja Reimann, Florian Settele, Manja Gloser-Bräunig, Erik Fiedler and Christoph Parthier

Contents

Supplementary discussion 2
Supplementary Figure S1 5
Supplementary Figure S2 6
Supplementary Figure S3 7
Supplementary Figure S4 8
Supplementary Figure S5 9
Supplementary Figure S6 10
Supplementary Figure S7 11
Supplementary Figure S8 12
Supplementary Figure S9 13
Supplementary Figure S10 14
Supplementary Figure S11 15
Supplementary Figure S12 16
Supplementary Figure S13 17
Supplementary Figure S14 18
Supplementary Figure S15 19
Supplementary Table S1: Binding characteristics and thermal stabilities of Af2 variants 20
Supplementary references 21

Supplementary discussion

Contribution of individual mutations evolved during affinity maturation of Af2

Maturation of the parental variant Af2p, yielding Af2s (the Strep-tagged version of Af2), resulted in a further increase of affinity towards 67B89, as shown by the low nanomolar K_D values from ELISA and SPR analysis (Table 1, Figure1). Interestingly, the increase of affinity during maturation seems to arise mostly from a 5-10-fold lower off-rate k_{off} for Af2s/Af2 compared to Af2p, while the on-rate k_{on} is only moderately affected. The recovery of thermal stability by the increase of +9K in T_m (Table 1, Figure 1b) during maturation of Af2p to Af2 is a consequence of the three sequence alterations (two mutations P38Q, Y143F and one deletion of I78). To further address the contribution of the individual events, three Af2p variants (Af2p-P38Q, Af2p-Y143F and Af2p-Δ78I) were generated, purified and subjected to binding analysis by SPR (Supplementary Table 1, Supplementary Figure S3). All three individual sequence changes occurring in the maturation process seem to contribute to higher affinity (lower K_D), by increasing the rate of association (k_{on}), while shortening the linker between the two Ub domains by deletion of Ile78 leads also to a decrease of the dissociation rate (k_{off}). Apparently, from the three mutations Δ78I has the largest contribution to the gain of affinity. A shorter linker between Ub-N and Ub-C limits the distance and conformational freedom of the domains relative to each other. Presumably, with a restricted arrangement both Ub domains could act as a single, more rigid binding module, rather than to two flexibly tethered binding domains. The benefit would be reduced entropic costs of target binding and could lead to the observed gain of affinity of Af2 variants, larger than a sole avidity effect of two tethered Ub-domains would produce [2].

Detailed description of the Af2 target-binding interface

In the complex structure of Af2:7B8, Ub-C contributes the major part (interface II, 485 Å and III, 326 Å^2) to the target binding interface compared to Ub-N (interface I: 368 Å^2). From 7B8, only the EDB and the Fn8 domain contribute to the binding interface, predominantly the EDB (interfaces I + II) , the Fn8 domain (interface III) to a lesser extent, while the Fn7 domain appears not to be directly involved in the interaction (Figure 2a).

Interface I residues F4 and evolved H6 from β1 of Ub-N are in hydrophobic interaction to EDB residues I1296 and F1315 (by π-stacking), respectively (Figure 2b). The sidechains of Ub-N K66 (evolved, α2β5 loop) and H68 (non-evolved, β5) interact via hydrogen bonds to backbone atoms of EDB I1295 and I1296. The carboxyl moiety of EDB D1317 sidechain forms an intermolecular hydrogen bond to the backbone amide of Ub-N K66, while also electrostatic interaction between the sidechains of both residues are conceivable.

Key interactions of interface II (Figure 2c) comprise a salt bridge between the sidechains of EDB D1314 and Ub-C R142 (evolved) and hydrophobic interactions of EDB F1312 to evolved F143. Both Ub-C residues are located in the α2β5 loop. Also the guanidine moiety of Ub-C R149 forms (partially water-mediated) hydrogens bonds and a salt bridge to the sidechain of EDB E1329. Several further contacts contribute to the intermolecular bond network of sub-site II: e.g. van-der-Waals interactions between Ub-C W123 and the EF loop region around G1327 of EDB.

Interface III (Figure 2d) employs only non-evolved residues: R120 (β3 strand), A124 (β3β4 loop) and the residues V147 and R149 (β5 strand) interact via (partially water-mediated) hydrogen bonds to Y1433 and S410 of Fn8, respectively. Additionally R149 forms a salt bridge to E1434 (FG loop) of Fn8. The target 7B8 exhibits an overall acidic character (calculated pI=4.1) and reveals a negative electrostatic potential at the surface (Supplementary Figure S6), while the Affilin Af2 (calculated pI=5.6) displays a positive electrostatic potential at the binding interface to 7B8. This charge complementarity of the Af2:7B8 interface suggests a contribution of ionic interactions, adding to the

framework of intermolecular hydrogen bonds, hydrophobic and van-der-Waals interactions, involved in target binding.
Structures of artificial binding proteins targeting 7B8 have been reported also for Anticalins (lipocalin-derived artificial binding proteins) evolved against oncofetal fibronectin [4, 5]. In spite of their distinct binding interfaces, they bind 7B8 in a surprisingly similar target conformation via EDB and the Fn8 domain, as observed for Affilin Af2 (supplementary figure S7).

Detailed analysis of the -2 register-shift in the Ub-N domain of Af2

The -2 register-shift of Ub-N is stabilized by a set of intramolecular interactions: e.g. a hydrophobic cluster formed by W45, I61, L67 and L69. While this cluster can also be formed in the non-shifted conformation (with L65 and L67 in place of L67 and L69, respectively), a second hydrophobic cluster, formed by I44 and V70, the π-stacking interactions of F4 and H68 and an intramolecular hydrogen bond between H68 and the evolved residue H6 can only occur in the -2-shifted state (Supplementary Figure S11a). The non-shifted conformation would also be destabilized by the evolved charged residue D8 of Af2, precluding the hydrophobic interactions of L8 to V70, found in the structure of of Ub-wt.
The bound target contributes to further stabilization of the register-shifted conformation of Ub-N, e.g. by the electrostatic interactions (D1317 of 7B8 to evolved K66 of Af2 Ub-N) and hydrogen bonds (between the sidechain of H68 and the backbone carbonyl oxygen of I1296). These interactions can occur only in presence of the -2 register shift, bringing the respective residues into spatial proximity (Figure 2b).
The α2β5 loop extension, induced by the -2 register shift, could impose sterical problems to this region of Ub-N with potentially detrimental effects on Affilin stability and binding affinity. To assess this further, the variant Af2s-ΔDP-KS was generated. It comprises a deletion of two residues (D62 and P63) from the α2β5 loop, and two additional mutations to restore original Ub-wt residues: loop residue L65 is reverted to lysine and L67 back to serine (Supplementary Figure S1). Thus, K65 and S67 of Af2s-ΔDP-KS should occupy the original positions of K63 and S65 in Ub-wt, avoiding hydrophobic residues in the α2β5 loop and at the S65-binding pocket, while the -2 register shift of β5 should be preserved. Evolved K66 (T66 in Ub-wt) was not substituted, as it is involved in EDB binding. Variant Af2s-ΔDP-KS revealed a remarkable increase of thermal stabiliy (ΔT_m=+9 K) compared to Af2s, and binding analysis by SPR revealed an about 5-fold higher affinity to the target 67B89 (Supplementary Figures S9 and S10, Supplementary Table S1). The observed gain of affinity resulted predominantly from an increased association rate (k_{on}). The data suggest that the -2 register-shifted state of Af2 Ub-N can be further stabilized by shortening the extended α2β5 loop size and reversal of residue dislocation at β5. Unfortunately, efforts to crystallise the variant Af2s-ΔDP-KS remained unsuccessful.

Detailed analysis of the -4 register-shift in the Ub-C domain of Af2

The four evolved residues in the extended α2β5 loop of Af2 Ub-C stabilize the -4 shift by several intra- and intermolecular interactions (Supplementary Figure S11b). The sidechain of F66* is buried in the hydrophobic cluster of W45*, L69*, V70* (asterisks designating residue numbering corresponding to Ub-N). An intra-loop salt bridge (and two hydrogen bonds) between R65* and D63* and the locked sidechain of Y64* (Y141), being sandwiched between the Ub-N and Ub-C domains, restrict the conformation of the α2β5 loop, further stabilizing the -4 shifted conformation. Intermolecular interactions to the bound target 7B8 also promote the -4 register shift: the electrostatic interactions of R65* and R72* to EDB residues D1314 and E1329, respectively, and the hydrophobic interaction between F66* and F1312 of EDB all involve Ub-C residues in register-shifted positions (Figure 2c). As in Af2 Ub-N, the two hydrophobic grooves of Ub-C are occupied by leucines, but now by L71* and L73* (Supplementary Figure 8d). The β5 strand terminates at R72* (located at the position of H68 in Ub-

wt), leaving a potential third binding site, harboring L71 in Ub-wt and L73 in Ub-N, unoccupied. To assess the relevance of a third leucine binding groove, two Af2s variants were generated: Af2s-GL and Af2s-AL. In both variants A75* is replaced by L75*, which could re-occupy the potential third site with a leucine (Supplementary Figure S1). Furthermore, in both variants R74* was replaced with either G74* or A74*, respectively, to reduce any potential sterical restrictions from the bulky side chain of Arg in its shifted position. Thermal stabilities of the Af2 variants and binding to the target 67B89 were analysed (Supplementary Table S1, Supplementary Figures S9 and S10). The amino acid changes showed only moderate effects: while the thermal stabilities were decreased (Af2s-GL by 2 K and Af2s-AL by 8 K), the target binding affinities remained almost unchanged, in spite of higher rates of association (k_{on}) and dissociation (k_{off}). This defines only the L67/L69-binding grooves of ubiquitin to be relevant, allowing register shifts by 2 or 4 residues. In all three states of β5 (non-shifted, -2-shifted, -4-shifted), both leucine-binding grooves are occupied by leucines residues (Supplementary Figure 8c,d).

Interestingly, the extension of the α2β5 loop of Af2 Ub-C by four residues from β5 coincides with the deletion of a residue (ΔQ140) from the same loop during the evolution of Af2 by phage display, resulting in an extension of α2β5 by only three residues (Supplementary Figure S1). Presumably, the deletion counteracted the -4 register shift, by partially compensating the space requirements of the four additional amino acids to be accommodated in the loop. Similar findings were previously reported for a mono-ubiquitin variant (Ubv-10F), evolved to bind tumor necrosis factor TNFα [6]. Two amino acids (D58, Y59) at the N-terminal boundary of the α2β5 loop region were found to be deleted during evolutionary selection of Ubv-10F. Re-introduction of both residues at their original location abolished binding of the ubiquitin variant to the target TNFα. However, without a three-dimensional structure of Ubv-10F it can only be speculated, that a β5 register shift is also present in Ubv-10F, defining a prerequisite for target binding.

Detailed analysis of the -2 register-shifts in Ub-N and Ub-C of Af1

The overall domain structures of Af1 Ub-N und Ub-C are very similar to each other. Also the α2β5 loops of both domains adopt a nearly identical backbone conformation, although they comprise different sets of evolved residues (Figure 4c). Interactions stabilising the -2 shift in Ub-N and Ub-C of Af1 strongly resemble also the interactions supporting the -2 register shift in Af2 Ub-N. The evolved residues L65 (Ub-N) and A65* (Ub-C) are displaced from their original position in β5, now inhabited by L67, contributing to the hydrophobic interactions clustered around W45 (involving also I61 and L69, identical residues in Ub-C not mentioned). V70, now -2-shifted to the former position of H68 in β5, forms an additional hydrophobic patch with I44 on the opposite face of the β-sheet, while H68 could form π-stacking interactions to an aromatic residue (W4, F4*) in the neighbouring β1 strand. Interestingly, for both Ub domains of Af1 the register shifts appear to be stabilized by chelation of copper ions from the crystallisation solution, which coordinate the imidazole side chains of H6 (β1) and H68 (β5), while in Ub-N of Af2 the same residues share a hydrogen bond instead (Supplementary Figure S12).

Supplementary Figure S1

a

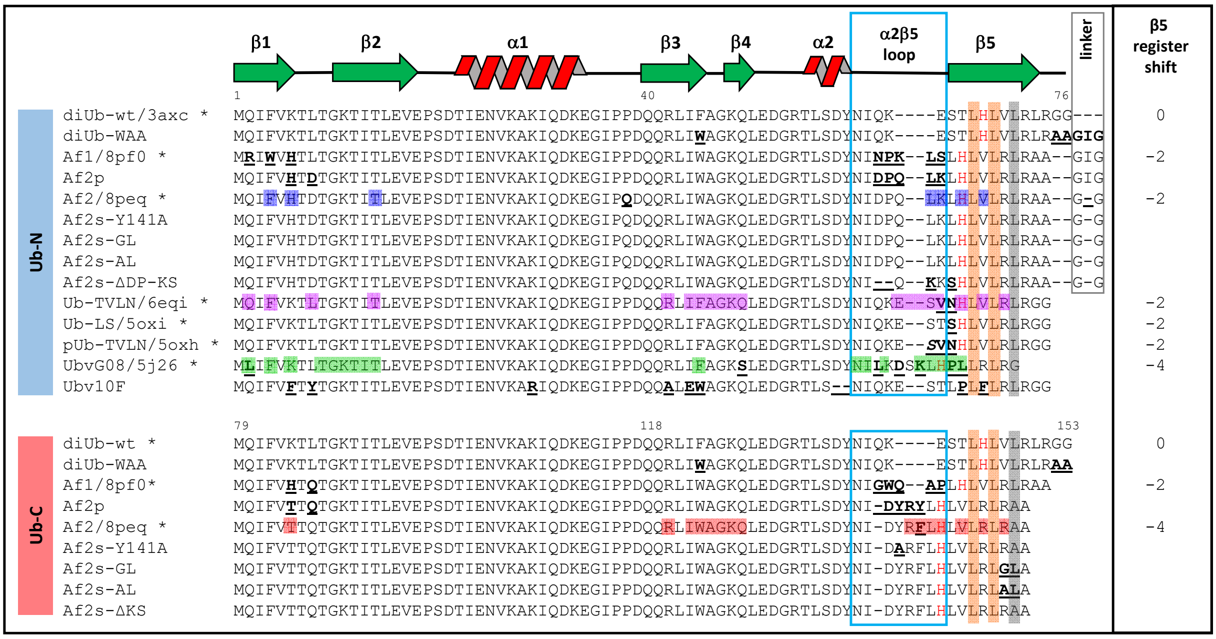

b

Af2-IL-2:

MQIFVHTDTGKTITLEVEPSDTIENVKAKIQDKEGIPQDQQRLIWAGKQLEDGRTLSDYNIDPQLKLHLVLRLRAAGG
MQIFVTTQTGKTITLEVEPSDTIENVKAKIQDKEGIPPDQQRLIWAGKQLEDGRTLSDYNIDYRFLHLVLRLRAA<u>SSS</u>
<u>SGSSSSGSSSSG</u>*APTSSSTKKTQLQLEHLLLDLQMILNGINNYKNPKLTRMLTFKFYMPKKATELKHLQCLEEELKPL*
EEVLNLAQSKNFHLRPRDLISNINVIVLELKGSETTFMCEYADETATIVEFLNRWITFSQSIISTLT

Figure S1: (a) Structure-based sequence alignment of diubiquitin-based Affilin variants, diubiquitin (diUb) and selected (mono) ubiquitin variants (availability of experimental structure marked with an asterisk and PDB accession code). Upper panel: alignment of N-terminal Ub domains (or mono-Ub domains), lower panel: alignment of C-terminal Ub domains. Residue numbering and secondary structure assignment according to Af2. Sequence alterations to respective parental Affilin variant (or Ub-wt) indicated in bold and underlined (except linker residues). Residues located in the α2β5 loop are boxed in blue, linker residues in grey box, positions of Leu residues in β5 of Ub-wt shaded in orange and grey, residue H68 colored in red. Residues identified in the complex structures involved in binding are colour-shaded. Register shifts in β5, if observed, given in the right column. The C-terminal Strep-tag II (SAWSHPQFEK) of Af2p, Af2s and their derived variants is not shown. **(b)** Amino acid sequence of the Af2-Interleukin-2 fusion (Af2-IL-2): human Interleukin-2 (C125S variant, italic letters) was genetically fused to the C-terminus of Af2, connected by a $(Ser_4\text{-}Gly)_3$ linker (underlined letters).

Supplementary Figure S2

67B89	------MGVFTTLQPGSSIPPYNTEVTETTIVITWTPAPRIGFKLGVRPSQGGEAPREVT
67B	*MHHHHHH*GVFTTLQPGSSIPPYNTEVTETTIVITWTPAPRIGFKLGVRPSQGGEAPREVT
7B8	--
B89	--
6789	------MGVFTTLQPGSSIPPYNTEVTETTIVITWTPAPRIGFKLGVRPSQGGEAPREVT
67B89	SDSGSIVVSGLTPGVEYVYTIQVLRDGQERDAPIVNKVVTPLSPPTNLHLEANPDTGVLT
67B	SDSGSIVVSGLTPGVEYVYTIQVLRDGQERDAPIVNKVVTPLSPPTNLHLEANPDTGVLT
7B8	--MLSPPTNLHLEANPDTGVLT
B89	--
6789	SDSGSIVVSGLTPGVEYVYTIQVLRDGQERDAPIVNKVVTPLSPPTNLHLEANPDTGVLT
67B89	VSWERSTTPDITGYRITTTPTNGQQGNSLEEVVHADQSSSTFDNLSPGLEYNVSVYTVKD
67B	VSWERSTTPDITGYRITTTPTNGQQGNSLEEVVHADQSSSTFDNLSPGLEYNVSVYTVKD
7B8	VSWERSTTPDITGYRITTTPTNGQQGNSLEEVVHADQSSSTFDNLSPGLEYNVSVYTVKD
B89	--
6789	VSWERSTTPDITGYRITTTPTNGQQGNSLEEVVHADQSSSTFDNLSPGLEYNVSVYTVKD
67B89	DKESVPISDTIIP**EVPQLTDLSFVDITDSSIGLRWTPLNSSTIIGYRITVVAAGEGIPIF**
67B	DKESVPISDTIIP**EVPQLTDLSFVDITDSSIGLRWTPLNSSTIIGYRITVVAAGEGIPIF**
7B8	DKESVPISDTIIP**EVPQLTDLSFVDITDSSIGLRWTPLNSSTIIGYRITVVAAGEGIPIF**
B89	------------M**EVPQLTDLSFVDITDSSIGLRWTPLNSSTIIGYRITVVAAGEGIPIF**
6789	DKESVPISDTIIPA--
67B89	**EDFVDSSVGYYTVTGLEPGIDYDISVITLINGGESAPTTLTQQTAVP**PPTDLRFTNIGPD
67B	**EDFVDSSVGYYTVTGLEPGIDYDISVITLINGGESAPTTLTQQTAVP**-------------
7B8	**EDFVDSSVGYYTVTGLEPGIDYDISVITLINGGESAPTTLTQQTAVP**PPTDLRFTNIGPD
B89	**EDFVDSSVGYYTVTGLEPGIDYDISVITLINGGESAPTTLTQQTAVP**PPTDLRFTNIGPD
6789	---VPPPTDLRFTNIGPD
67B89	TMRVTWAPPPSIDLTNFLVRYSPVKNEEDVAELSISPSDNAVVLTNLLPGTEYVVSVSSV
67B	--
7B8	TMRVTWAPPPSIDLTNFLVRYSPVKNEEDVAELSISPSDNAVVLTNLLPGTEYVVSVSSV
B89	TMRVTWAPPPSIDLTNFLVRYSPVKNEEDVAELSISPSDNAVVLTNLLPGTEYVVSVSSV
6789	TMRVTWAPPPSIDLTNFLVRYSPVKNEEDVAELSISPSDNAVVLTNLLPGTEYVVSVSSV
67B89	YEQHESTPLRGRQKTGLDSPTGIDFSDITANSFTVHWIAPRATITGYRIRHHPEHFSGRP
67B	--
7B8	YEQHESTPLRGRQKT---
B89	YEQHESTPLRGRQKTGLDSPTGIDFSDITANSFTVHWIAPRATITGYRIRHHPEHFSGRP
6789	YEQHESTPLRGRQKTGLDSPTGIDFSDITANSFTVHWIAPRATITGYRIRHHPEHFSGRP
67B89	REDRVPHSRNSITLTNLTPGTEYVVSIVALNGREESPLLIGQQSTV--------
67B	--
7B8	--
B89	REDRVPHSRNSITLTNLTPGTEYVVSIVALNGREESPLLIGQQSTV*LEHHHHHH*
6789	REDRVPHSRNSITLTNLTPGTEYVVSIVALNGREESPLLIGQQSTV--------

Figure S2. Sequence alignment of fibronectin fragments used in this work. Derived from human fibronectin isoform 7 containing the EDB (UniProtKB database ID: P02751, amino acids 1080 to 1541), several truncated constructs were generated for selection and maturation of binders (target 67B89, off-target 6789 lacking EDB), binding analysis (all variants) and crystallisation (7B8). EDB residues shown with bold letters, additional residues from purification tags as italic letters.

Supplementary Figure S3

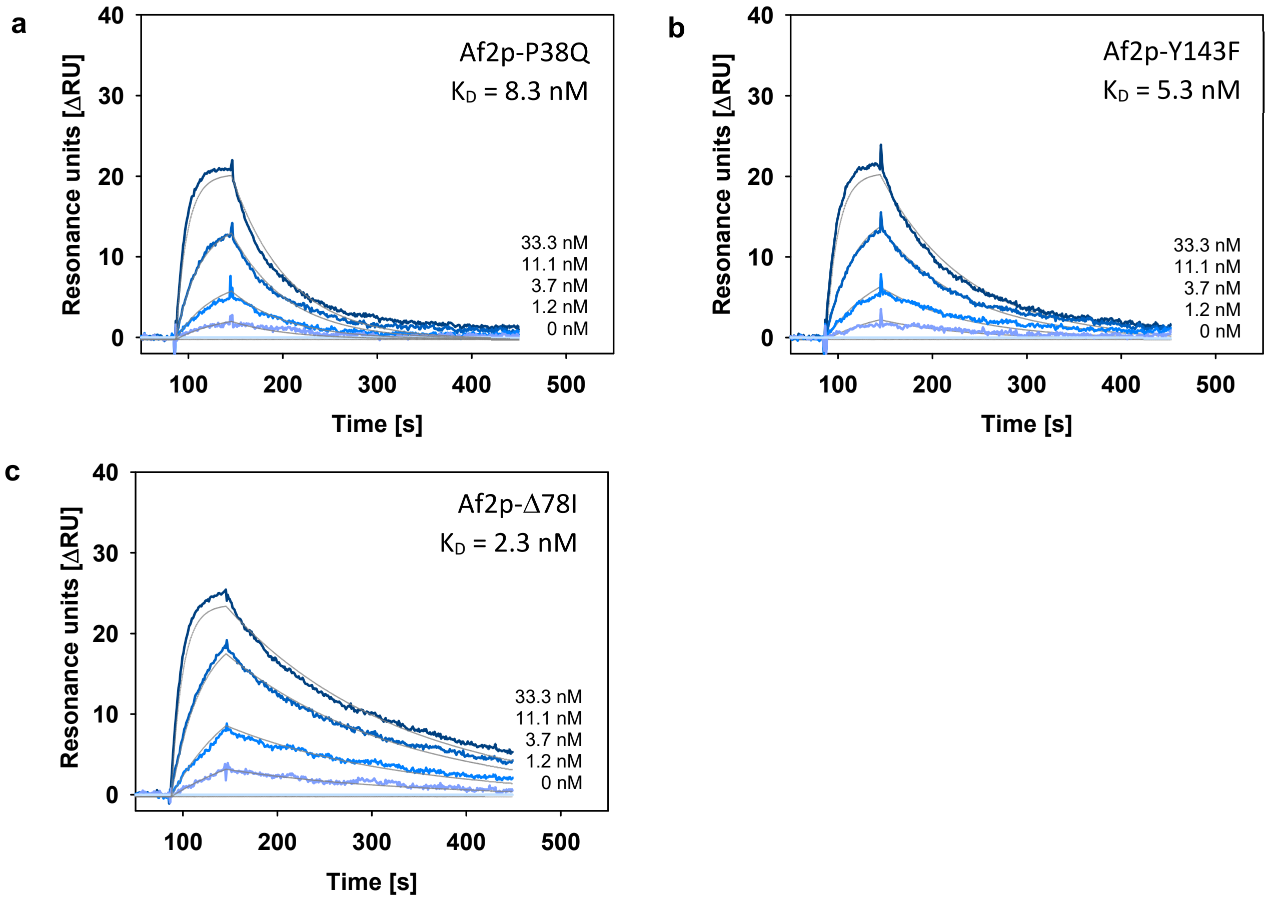

Figure S3. SPR analysis of Affilin variants generated during the directed evolution process binding to the immobilized target 67B89 (complete binding parameters are given in Supplementary Table 1). (**a**) and **(b)** single amino acid exchange variants and **(c)** deletion mutant of Af2p. Each variant comprises one of the three mutations acquired during affinity maturation of Af2p to study their individual contribution.

Supplementary Figure S4

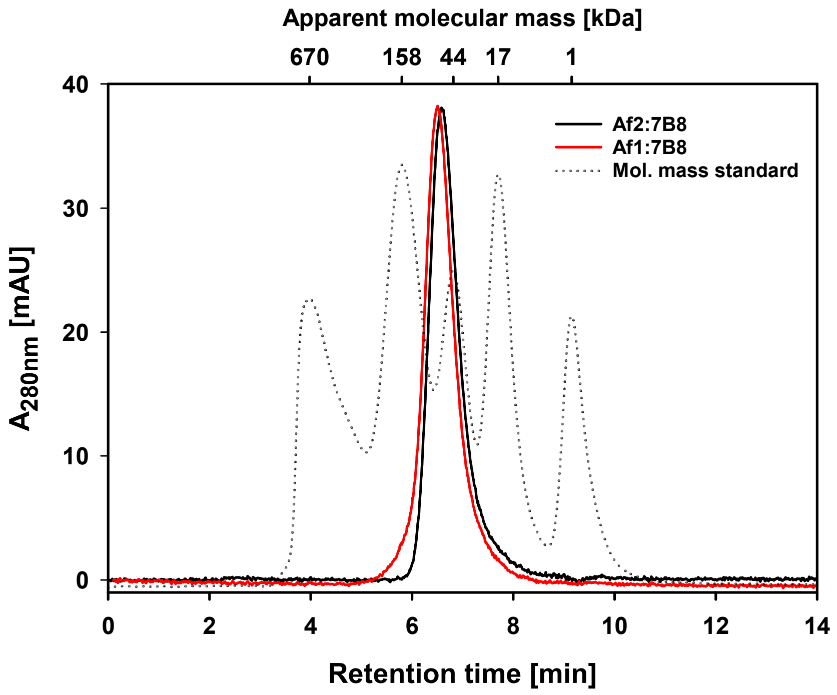

Figure S4. Size exclusion HPLC analysis of the Af2:7B8 and Af1:7B8 complexes. The preequilibrated complexes were analyzed using a Superdex 200 5/150 GL analytical size exclusion column at a flow rate of 0.3 ml/min. The column was calibrated with a HPLC gel filtration molecular weight standard consisting of Thyroglobulin (670 kDa), Gamma-globulin (158 kDa), Ovalbumin (44 kDa), Myoglobin (17 kDa) and vitamin B12 (1 kDa). Apparent molecular weights were derived from the retention times of the Af2:7B8 complex (black solid line) and the Af1:7B8 complex (red solid line), respectively, using the molecular weight standard (dotted line).

Supplementary Figure S5

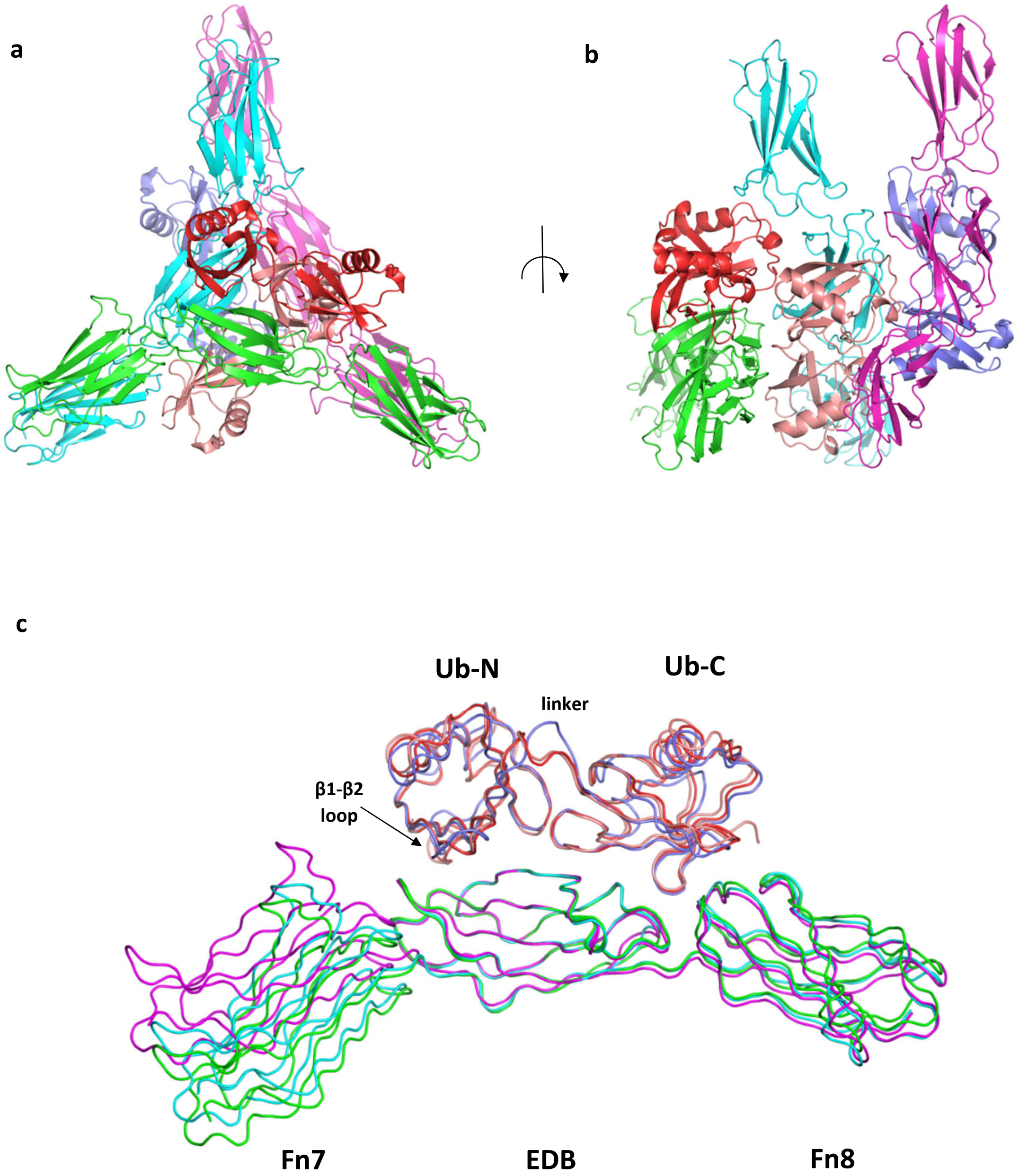

Figure S5. Asymmetric unit of the Af2:7B8 complex crystal structure composed by three complexes. (**a**) 3_1-fold non-crystallographic symmetry relation of the three complexes colored by chain (7B8 : chain A – green, chain B – cyan, chain C – magenta, Af2 : chain L – red, chain J – salmon, chain M – blue) **(b)** 90° rotated view (**c**)**:** Ribbon representation of a structural comparison of the three Af2:7B8 complexes from the asymmetric unit. For superposition, only the EDB residues (1266 – 1356) of the 7B8 chains were aligned.

Supplementary Figure S6

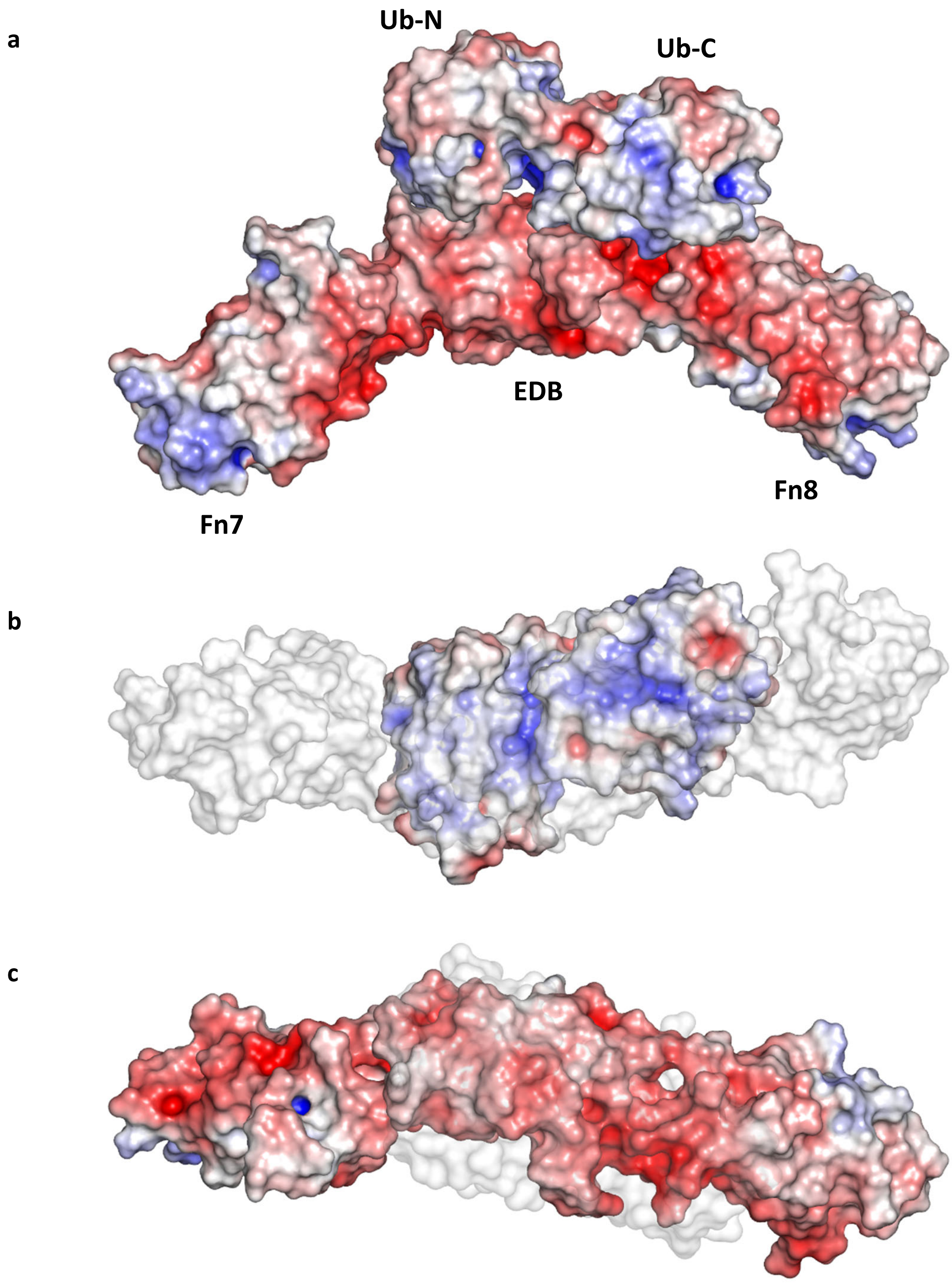

Figure S6. Electrostatic properties of Af2 and 7B8. ±5 kT/e electrostatic potential APBS Electrostatic surface potentials were calculated using the software APBS [7] with the non-linear Poisson-Boltzmann equation contoured at 5 kT/e. Negatively and positively charged surface areas are colored in red and blue, respectively. (**a**) Side view in the orientation of Figure 2a. (**b**) Rotated view on the 7B8-binding interface of Af2, the bound target 7B8 is schown as transparent white surface. (c) Rotated view on the Af2-binding face of 7B8, bound Af2 is shown as transparent white surface.

Supplementary Figure S7

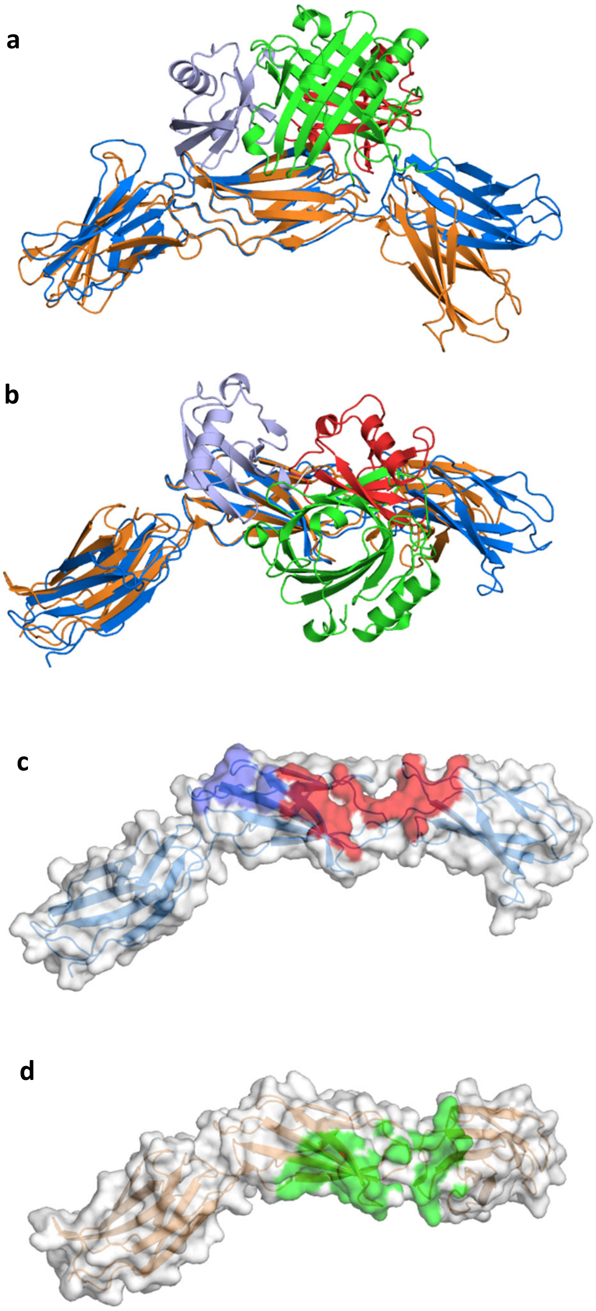

Figure S7. Structural comparison of the Af2:7B8 complex and 7B8-bound Anticalin N7A (PDB id 4GH7 [4]) (**a**) Superposition of overall structures based on alignment of the EDBs shown in the orientation of Figure 2a. Af2 colored in light blue (Ub-N) and red (Ub-C), Af2-bound 7B8 colored in blue, N7A colored im green, N7A-bound 7B8 colored in orange. (**b**) 90° rotated view. (**c**) Molecular surface of Af2-bound 7B8 with mapped binding interfaces of Ub-N (blue surface) and Ub-C (red surface). (**d**) Molecular surface of N7A-bound 7B8 with mapped binding interface of N7A (green surface).

Supplementary Figure S8

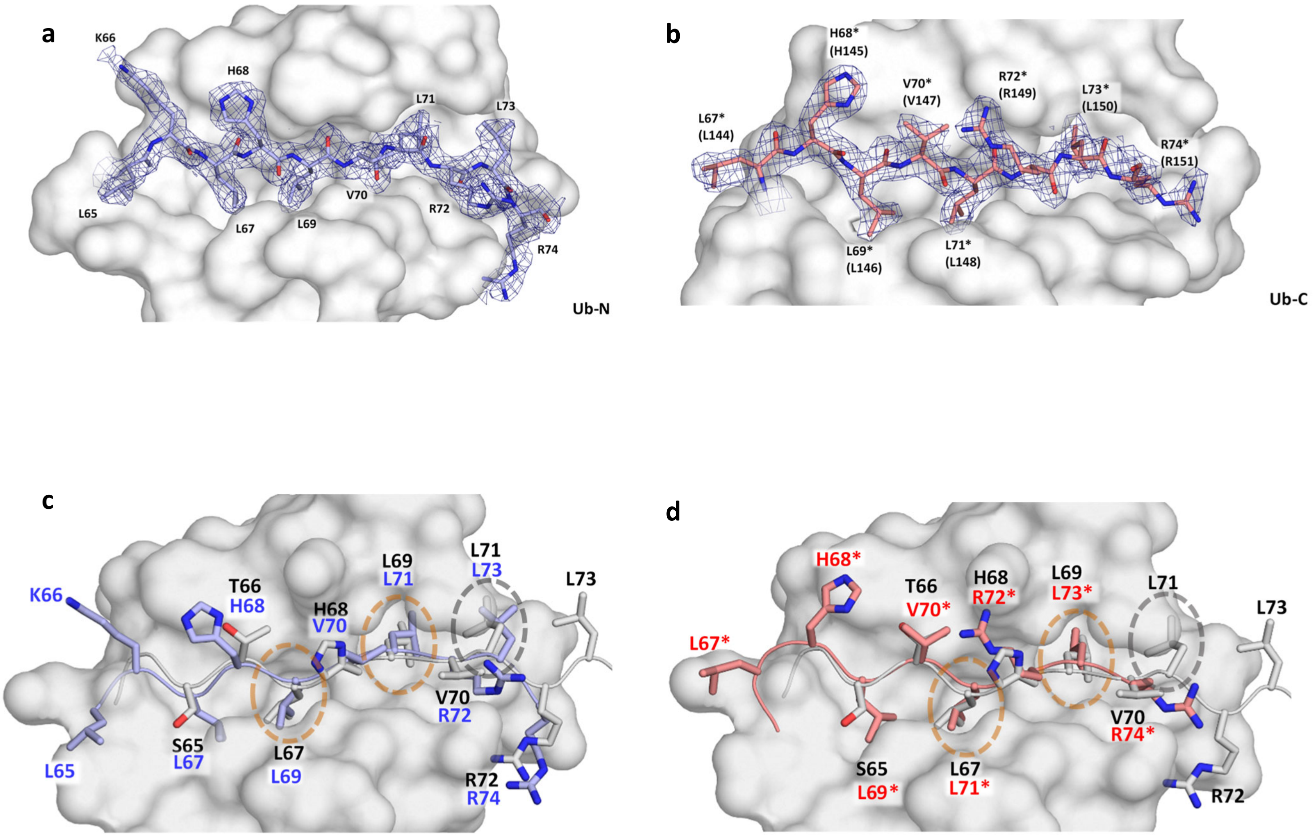

Figure S8. (**a**) 2F$_o$-F$_c$ electron density (blue mesh) countoured at 1σ of residues from the β5 strand of Af2 Ub-N, revealing a -2 register shift. Residues are shown as sticks, Ub-N residues (except from β5) shown as white surface. (**b**) 2F$_o$-F$_c$ electron density (blue mesh) countoured at 1σ of residues from the β5 strand of Af2 Ub-C, revealing a -4 register shift. Residues are shown as sticks, Ub-N residues (except from β5) shown as white surface. Residue labels marked with an asterisk corresponds to the residue numbers of Ub-N. (**c**) Structural superposition of residues from the β5 strand of Af2 Ub-N (light blue sticks, blue labels) and Ub-wt (PDB id 1UBQ [2], white sticks, black labels) depicting the -2 register shift. (**d**) Structural superposition of residues from the β5 strand of Af2 Ub-C (light red sticks, red labels) and Ub-wt (white sticks, black labels) depicting the -4 register shift. The two hydrophobic binding grooves harboring leucine residues in the non-shifted and -2/-4 register-shifted states are marked with orange circles. The grey circle marks a leucine-harboring site occupied only in the non-shifted and -2-shifted state.

Supplementary Figure S9

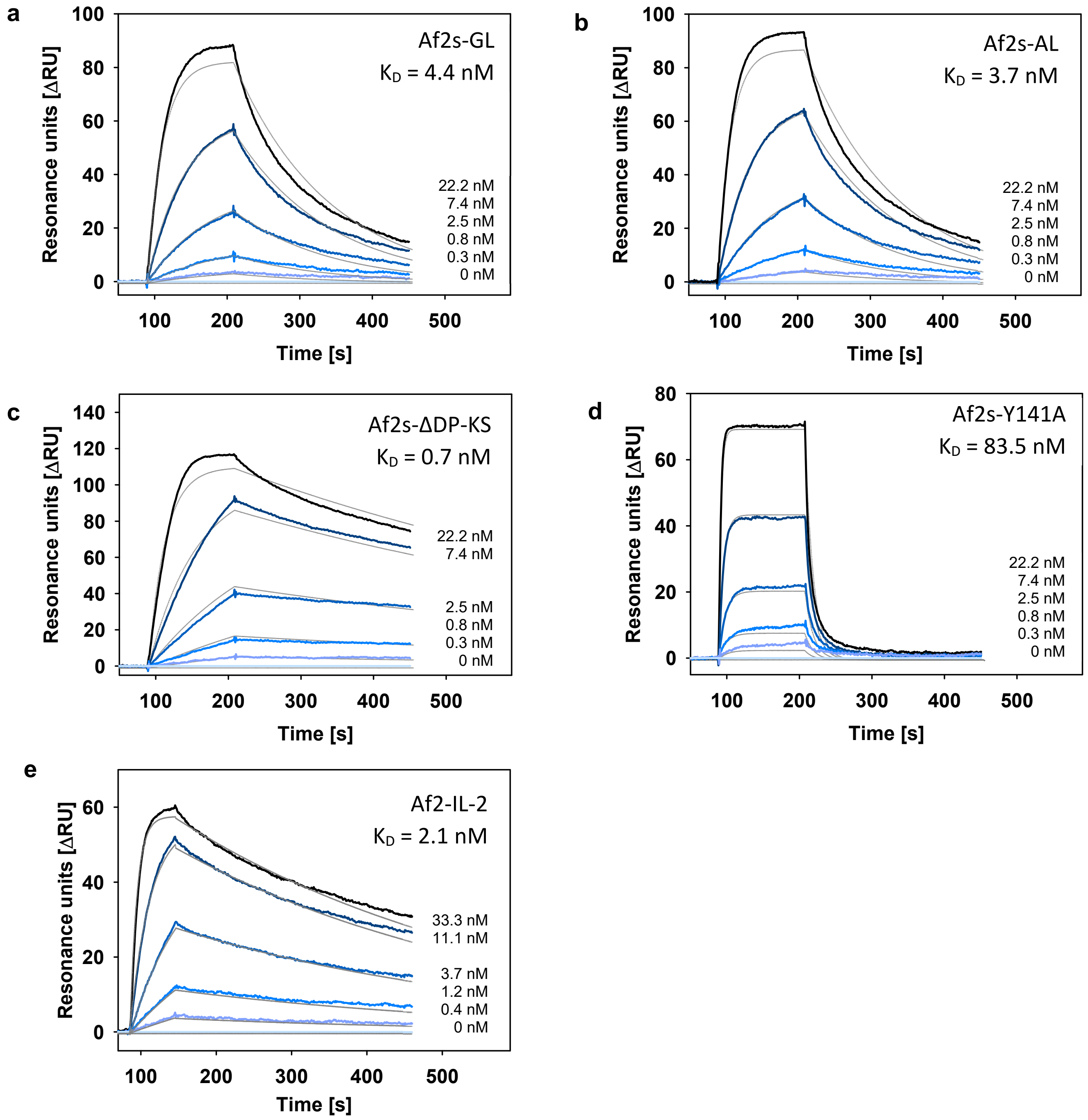

Figure S9. Influence of selected modifications on the target binding properties of Af2s analysed by SPR at different Affilin concentrations (complete binding parameters are given in Table 1 and Supplementary Table 1). (**a**) Variant Af2s-GL carrying the mutations R74*G and A75*L in Ub-C. (**b**) Variant Af2s-AL carrying the mutations R74*A and A75*L in Ub-C. (**c**) Variant Af2s-ΔDP-KS carrying the deletions ΔD62 and ΔP63 and the mutations L65K and L67S in Ub-N. (**d**) Variant Af2s-Y141A (Y64*A). (**e**) Variant Af2-IL-2, genetic fusion of Af2 to human IL-2.

Supplementary Figure S10

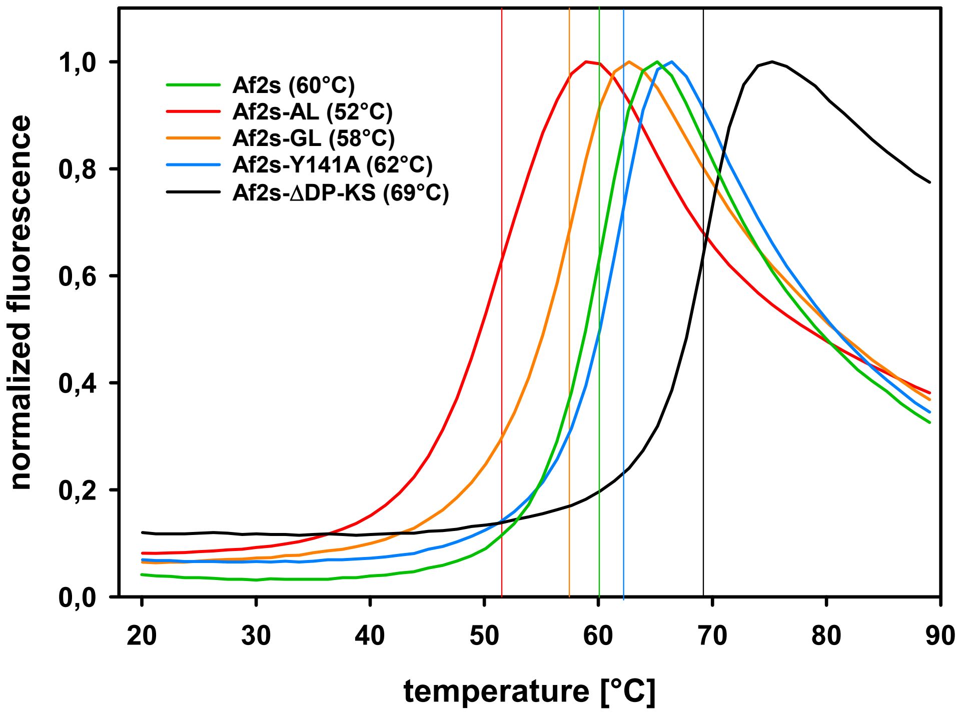

Figure S10. Analysis of thermal stabilities of Affilin variants by DSF. Variant Af2s-GL carrying the mutations R74*G and A75*L in Ub-C, variant Af2s-AL carrying the mutations R74*A and A75*L in Ub-C, variant Af2s-ΔDP-KS carrying the deletions ΔD62 and ΔP63 and the mutations L65K and L67S in Ub-N and variant Af2s-Y141A (Y64*A). Vertical lines mark the midpoints (T_m) of thermal unfolding. T_m values are given in Table1 and Supplementary Table 1, respectively.

Supplementary Figure S11

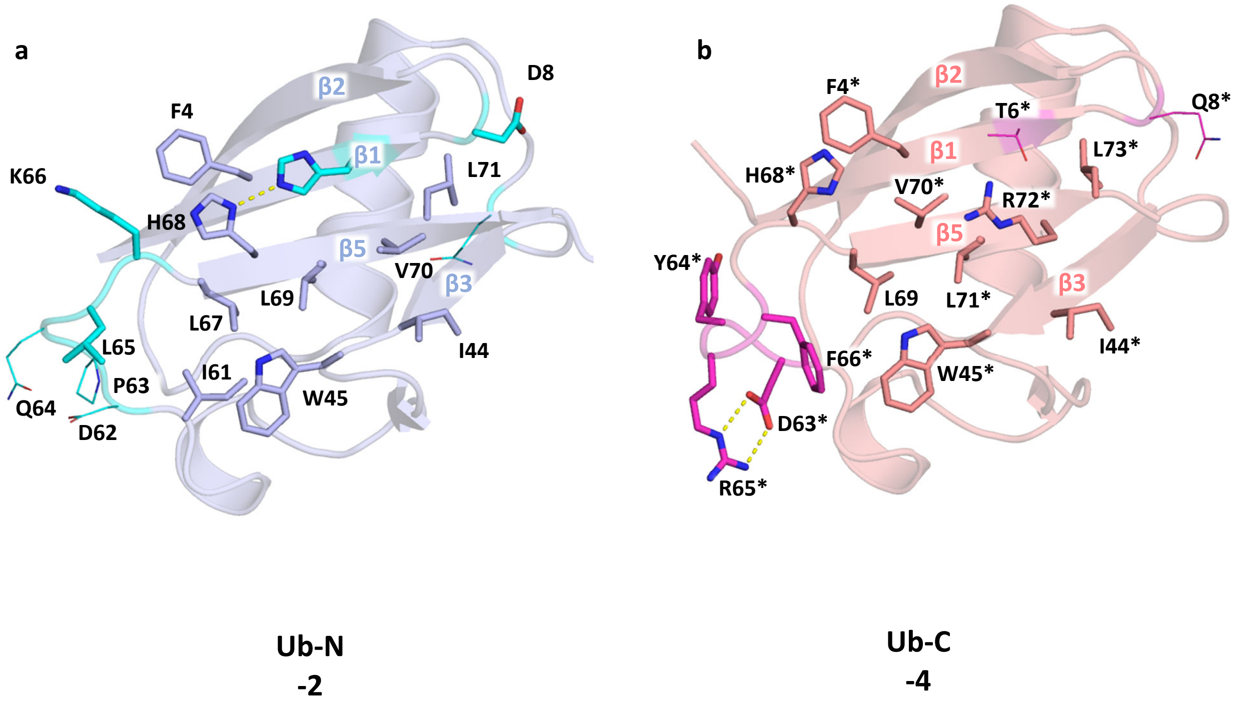

Figure S11. Intramolecular interactions stabilizing the register-shifted states observed in Af2. (**a**) Residues involved in stabilizing the -2 shift in Ub-N shown as sticks, evolved amino acids colored in cyan, shown as lines if not involved in intramolecular stabilization of the shift. (**b**) Residues involved in stabilizing the -4 register shift in Ub-C shown as sticks, evolved amino acids colored in magenta, shown as lines if not involved in intramolecular stabilisation of the shift. Residue labels marked with an asterisk corresponds to the residue numbering of Ub-N.

Supplementary Figure S12

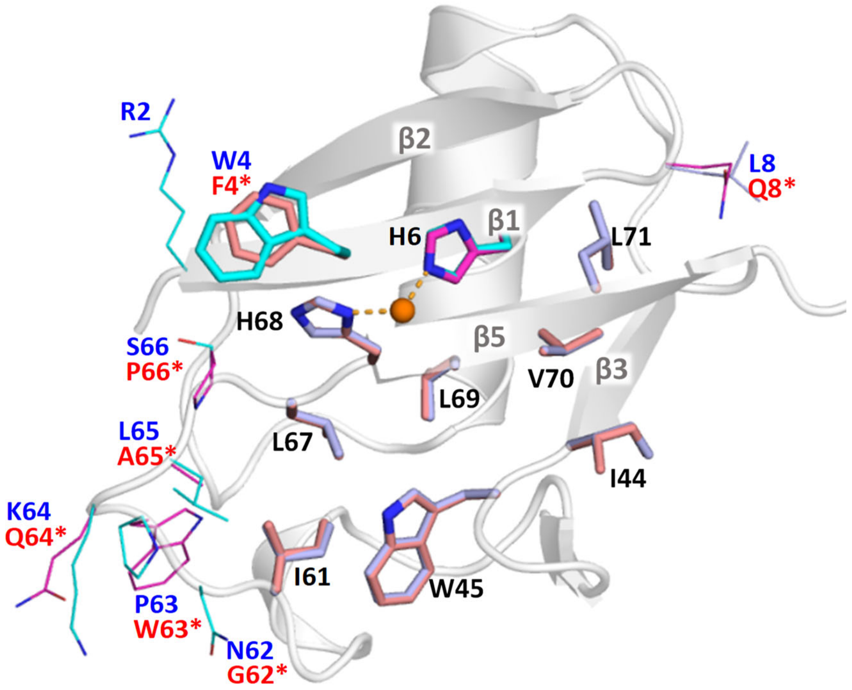

Ub-N / Ub-C
-2 / -2

Figure S12. The -2 register shifts observed in the crystal structure of unbound Af1. Superimposed residues of Ub-N and Ub-C involved intramolecular stabilization of the -2 shift in Af1, shown as sticks, evolved amino acids colored in cyan (Ub-N, blue labels) and magenta (Ub-C, red labels). Residues shown as lines if not involved in intramolecular stabilization of the shift. Residue labels marked with an asterisk corresponds to the residue numbering of Ub-N, residues are identical in Ub-N and Ub-C are labelled in black. H68 and H6 of Ub-N and Ub-C coordinate a copper ion (orange sphere and dashes) present in the crystallisation solution.

Supplementary Figure S13

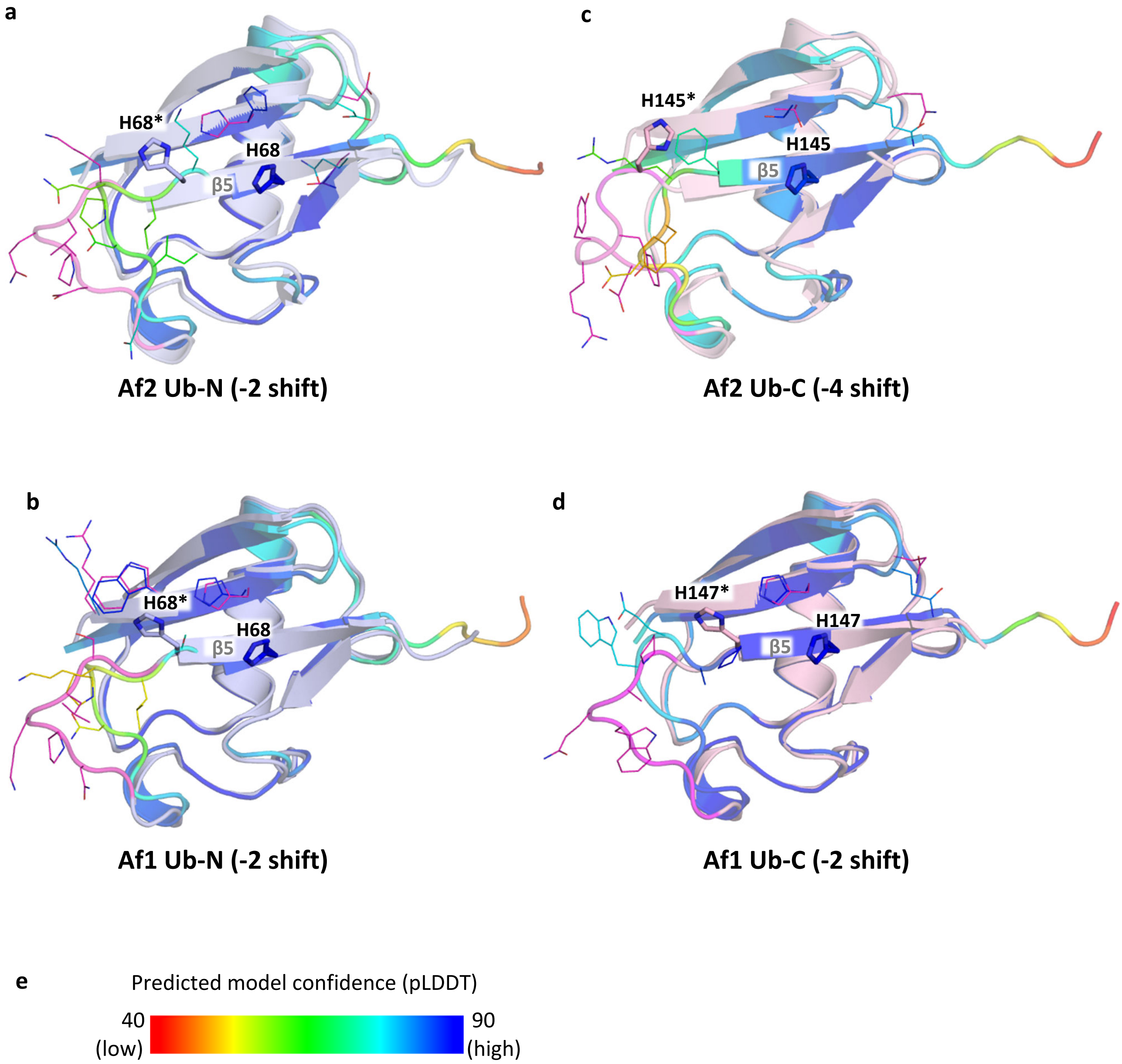

Figure S13. Comparison of experimentally determined structures of Affilin Ub-N and Ub-C domains with hypothetical models generated by Alphafold2 [8]. Experimental structures of the individual Ub-N domains of **(a)** Af2 and **(b)** Af1, coloured in light blue ($\alpha 2\beta 5$ loop colored in magenta), were superimposed to the respective predicted structural models (colored by predicted model confidence, pLDDT score). Side chains of evolved residues shown as lines, side chain of H68 shown as sticks, indicating the register shifts. Superposition of the individual Ub-C domains of **(c)** Af2 and **(d)** Af1, respectively: experimental structures are shown in light pink ($\alpha 2\beta 5$ loop colored in magenta), predicted Ub-C structures colored according to predicted model confidence (pLDDT). Side chains of evolved residues shown as lines, side chains of H145 (Af2) and H147 (Af1) shown as sticks. None of the register shifts of Af1 and Af2 (indicated in parentheses, residue labels with asterisk) were observed in the predicted models. **(e)** Color scale used for the hypothetical Ub-N/Ub-C structures, based on the Alphafold2 model's prediction of its score on the local Distance Difference Test (pLDDT), from low confidence (red) to high confidence (blue).

Supplementary Figure S14

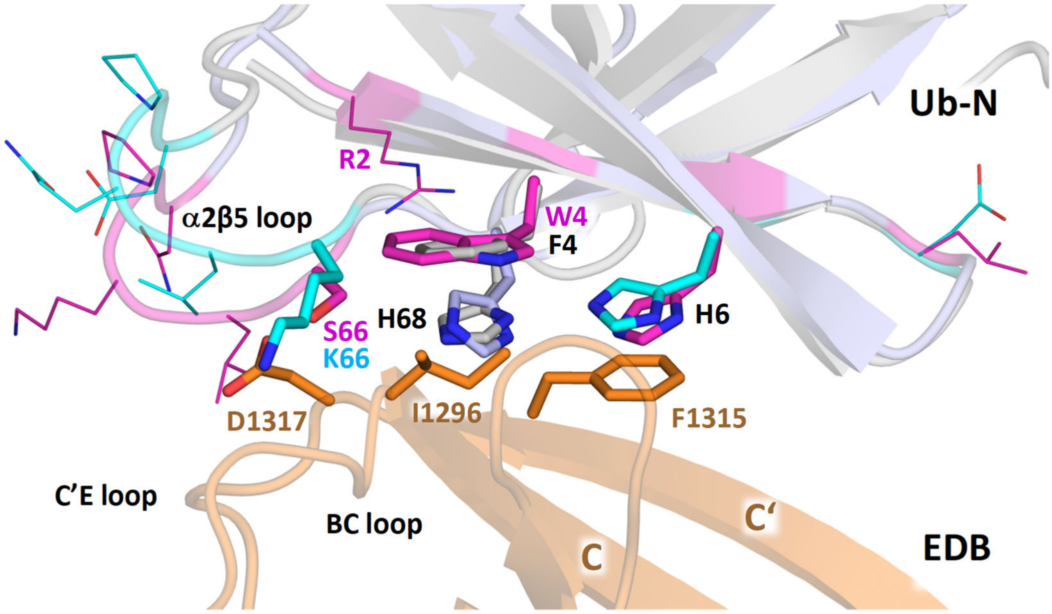

Figure S14. Structural superposition of the Ub-N domains of target-bound Af2 (Ub-N colored in grey, evolved side chains in cyan, EDB in orange) and unbound Af1 (colored in light blue, evolved side chains in magenta). Residues of the binding interface I in the Af2:7B8 complex and corresponding residues in Af1 shown as sticks, other evolved residues shown as lines.

Supplementary Figure S15

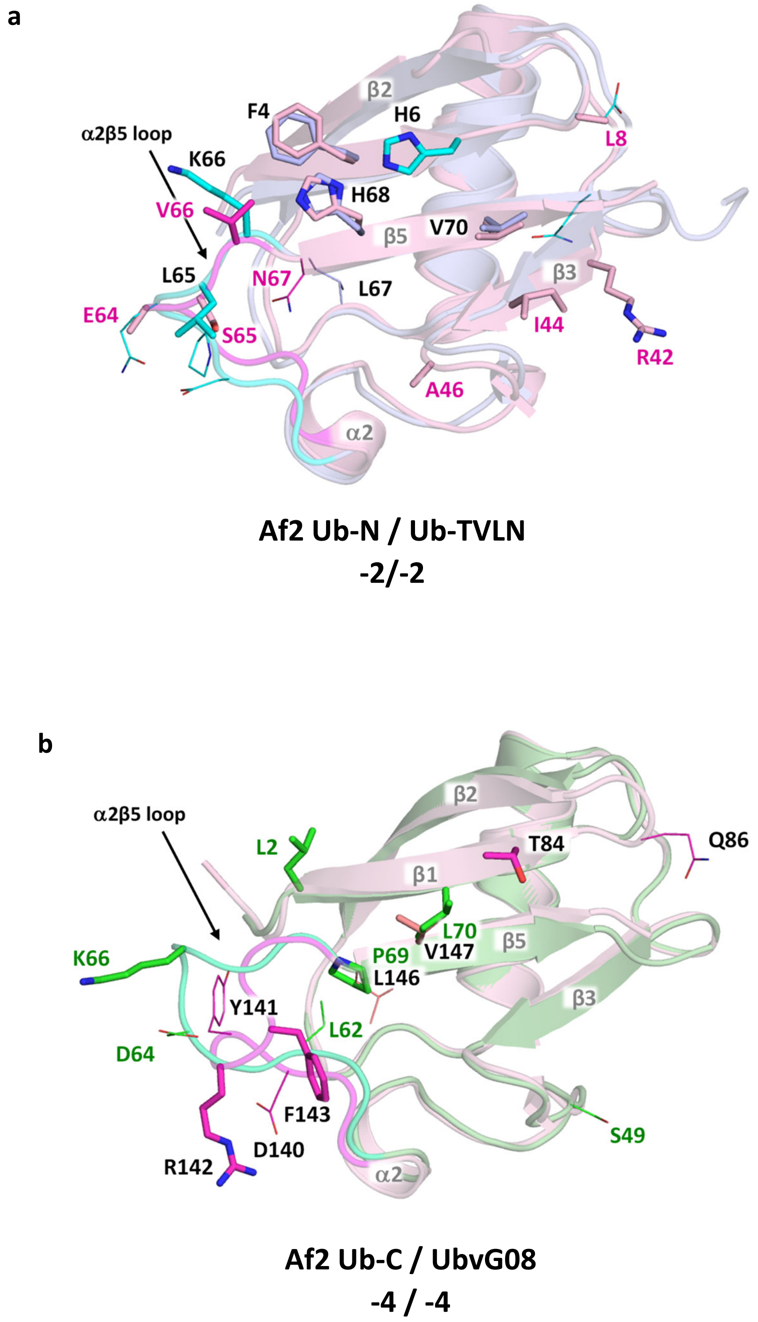

Figure S15. Structural comparison of the Af2:7B8 complex with other Ub variants exhibiting register shifts in the β5 strand. (**a**) Structural superposition of Af2 Ub-N (light blue, black labels, evolved residues and α2β5 loop colored in cyan) and Ub-TVLN (PDB id 6EQI [1], light red, magenta labels, α2β5 loop residues colored in magenta), both displaying a -2 register-shift in β5. Residues involved in binding shown as sticks. Altered residues not contributing to binding shown as lines. **(b)** Structural superposition of Af2 Ub-C (light red, black labels, evolved residues and α2β5 loop colored in magenta) and UbvG08 (PDB id 5J26 [3], light green, green labels, α2β5 loop residues colored in cyan), both displaying a -4 register-shift in β5. Residues involved in binding shown as sticks. Evolved residues not contributing to binding shown as lines.

Supplementary Table S1: Binding characteristics and thermal stabilities of Af2 variants

Affilin [1]	DSF T_m [°C]	ELISA K_D [nM]	SPR K_D [nM]	SPR k_{on} [1/M·s]	SPR k_{off} [1/s]
Af2p	53	7.7	17.1	$5.7 \cdot 10^{5}$	$9.8 \cdot 10^{-3}$
Af2p-P38Q	- [2]	- [2]	8.3	$2.0 \cdot 10^{6}$	$1.7 \cdot 10^{-2}$
Af2p-Y143F	- [2]	- [2]	5.3	$2.1 \cdot 10^{6}$	$1.1 \cdot 10^{-2}$
Af2p-Δ78I	- [2]	- [2]	2.3	$2.7 \cdot 10^{6}$	$5.5 \cdot 10^{-3}$
Af2s	60	3.1	3.7	$3.0 \cdot 10^{5}$	$1.1 \cdot 10^{-3}$
Af2s-GL	58	- [2]	4.4	$1.7 \cdot 10^{6}$	$7.6 \cdot 10^{-3}$
Af2s-AL	52	- [2]	3.7	$2.1 \cdot 10^{6}$	$7.9 \cdot 10^{-3}$

[1] binding to target 67B89
[2] not measured

Supplementary references

1. Schubert, A.F. et al. Structure of PINK1 in complex with its substrate ubiquitin. *Nature* **552**, 51-56 (2017).
2. Leung, I., Jarvik, N. & Sidhu, S.S. A Highly Diverse and Functional Naive Ubiquitin Variant Library for Generation of Intracellular Affinity Reagents. *J Mol Biol* **429**, 115-127 (2017).
3. Canny, M.D. et al. Inhibition of 53BP1 favors homology-dependent DNA repair and increases CRISPR–Cas9 genome-editing efficiency. *Nature Biotechnology* **36**, 95-102 (2018).
4. Gebauer, M., Schiefner, A., Matschiner, G. & Skerra, A. Combinatorial Design of an Anticalin Directed against the Extra-Domain B for the Specific Targeting of Oncofetal Fibronectin. *Journal of Molecular Biology* **425**, 780-802 (2013).
5. Schiefner, A., Gebauer, M., Richter, A. & Skerra, A. Anticalins Reveal High Plasticity in the Mode of Complex Formation with a Common Tumor Antigen. *Structure* **26**, 649-656.e643 (2018).
6. Hoffmann, A. et al. New binding mode to TNF-alpha revealed by ubiquitin-based artificial binding protein. *PLoS One* **7**, e31298 (2012).
7. Jurrus, E. et al. Improvements to the APBS biomolecular solvation software suite. *Protein Sci* **27**, 112-128 (2018).
8. Jumper, J. et al. Highly accurate protein structure prediction with AlphaFold. *Nature* **596**, 583-589 (2021).
