# Supplementary material for: Ubiquitin-derived artificial binding proteins targeting oncofetal fibronectin reveal scaffold plasticity by β-strand slippage
Source: Commun Biol. 2024 Jul 27;7:907. doi: 10.1038/s42003-024-06569-9 (PMC11283464; doi:10.1038/s42003-024-06569-9)
Supplement: Supplementary file 5 — Reporting summary [file 42003_2024_6569_MOESM5_ESM.pdf]

nature portfolio

Corresponding author(s): Christoph Parthier

Last updated by author(s): Apr 16, 2024

Reporting Summary

Nature Portfolio wishes to improve the reproducibility of the work that we publish. This form provides structure for consistency and transparency in reporting. For further information on Nature Portfolio policies, see our Editorial Policies and the Editorial Policy Checklist.

Statistics

For all statistical analyses, confirm that the following items are present in the figure legend, table legend, main text, or Methods section.

n/a	Confirmed	
☒	☐	The exact sample size (*n*) for each experimental group/condition, given as a discrete number and unit of measurement
☒	☐	A statement on whether measurements were taken from distinct samples or whether the same sample was measured repeatedly
☒	☐	The statistical test(s) used AND whether they are one- or two-sided<br>*Only common tests should be described solely by name; describe more complex techniques in the Methods section.*
☒	☐	A description of all covariates tested
☒	☐	A description of any assumptions or corrections, such as tests of normality and adjustment for multiple comparisons
☒	☐	A full description of the statistical parameters including central tendency (e.g. means) or other basic estimates (e.g. regression coefficient) AND variation (e.g. standard deviation) or associated estimates of uncertainty (e.g. confidence intervals)
☒	☐	For null hypothesis testing, the test statistic (e.g. *F*, *t*, *r*) with confidence intervals, effect sizes, degrees of freedom and *P* value noted<br>*Give P values as exact values whenever suitable.*
☒	☐	For Bayesian analysis, information on the choice of priors and Markov chain Monte Carlo settings
☒	☐	For hierarchical and complex designs, identification of the appropriate level for tests and full reporting of outcomes
☒	☐	Estimates of effect sizes (e.g. Cohen's *d*, Pearson's *r*), indicating how they were calculated

Our web collection on statistics for biologists contains articles on many of the points above.

Software and code

Policy information about availability of computer code

Data collection	X-ray diffraction oscillation images of Af1 crystals were recorded at beamline 14.2 at the BESSY synchrotron using the 'marccd' software package (Rayonix). X-ray diffraction images of Af2:7B8 complex crystals were recorded at BESSY BL14.1 using the 'MXCuBE 2.2 Qt4' software suite (European Synchrotron Radiation Facility).
Data analysis	X-ray diffraction data were processed with the XDS software package (version 11/11/2013). SAD-phasing of the Af1 crystal structure was carried out using the SHELX software package (shelxc, shelxd, shelxe version 2013/2). Heavy atom positions and phases were refined using the software SHARP (version 2.8.2) allowing automatic building of an initial Af1 model with the ARP/wARP software (version 7.3). The Af2:7B8 diffraction data were phased using the software PHASER (version 2.5.2) from the CCP4 suite (version 7.1). The structural models were manually completed using the program COOT (version 0.9.7) and refined with phenix.refine from the PHENIX software suite (version 1.20.1). Structure validation was carried out using MOLPROBITY from the PHENIX software suite. Molecular figures were created with the software PyMOL (version 2.5.2, Schrödinger LLC, New York, USA).

For manuscripts utilizing custom algorithms or software that are central to the research but not yet described in published literature, software must be made available to editors and reviewers. We strongly encourage code deposition in a community repository (e.g. GitHub). See the Nature Portfolio guidelines for submitting code & software for further information.

Data

Policy information about availability of data

All manuscripts must include a data availability statement. This statement should provide the following information, where applicable:

- Accession codes, unique identifiers, or web links for publicly available datasets
- A description of any restrictions on data availability
- For clinical datasets or third party data, please ensure that the statement adheres to our policy

The data availability statement has been placed in the manuscript. Data deposition: The atomic coordinates and structure factors of unbound Af1 and the Af2:7B8 complex have been deposited in the Protein Data Bank, www.pdb.org (PDB ID codes 8PF0 and 8PEQ, respectively) and will be released upon publication of the research article (HPUB).

Human research participants

Policy information about studies involving human research participants and Sex and Gender in Research.

Reporting on sex and gender	No human participants, their data or biological material were used in this study.
Population characteristics	No human participants, their data or biological material were used in this study.
Recruitment	No human participants, their data or biological material were used in this study.
Ethics oversight	No human participants, their data or biological material were used in this study.

Note that full information on the approval of the study protocol must also be provided in the manuscript.

Field-specific reporting

Please select the one below that is the best fit for your research. If you are not sure, read the appropriate sections before making your selection.

☒ Life sciences ☐ Behavioural & social sciences ☐ Ecological, evolutionary & environmental sciences

For a reference copy of the document with all sections, see nature.com/documents/nr-reporting-summary-flat.pdf

Life sciences study design

All studies must disclose on these points even when the disclosure is negative.

Sample size	*Describe how sample size was determined, detailing any statistical methods used to predetermine sample size OR if no sample-size calculation was performed, describe how sample sizes were chosen and provide a rationale for why these sample sizes are sufficient.*
Data exclusions	*Describe any data exclusions. If no data were excluded from the analyses, state so OR if data were excluded, describe the exclusions and the rationale behind them, indicating whether exclusion criteria were pre-established.*
Replication	*Describe the measures taken to verify the reproducibility of the experimental findings. If all attempts at replication were successful, confirm this OR if there are any findings that were not replicated or cannot be reproduced, note this and describe why.*
Randomization	*Describe how samples/organisms/participants were allocated into experimental groups. If allocation was not random, describe how covariates were controlled OR if this is not relevant to your study, explain why.*
Blinding	*Describe whether the investigators were blinded to group allocation during data collection and/or analysis. If blinding was not possible, describe why OR explain why blinding was not relevant to your study.*

Reporting for specific materials, systems and methods

We require information from authors about some types of materials, experimental systems and methods used in many studies. Here, indicate whether each material, system or method listed is relevant to your study. If you are not sure if a list item applies to your research, read the appropriate section before selecting a response.

Materials & experimental systems

n/a	Involved in the study	
☐	☒	Antibodies
☐	☒	Eukaryotic cell lines
☒	☐	Palaeontology and archaeology
☒	☐	Animals and other organisms
☒	☐	Clinical data
☒	☐	Dual use research of concern

Methods

n/a	Involved in the study	
☒	☐	ChIP-seq
☒	☐	Flow cytometry
☒	☐	MRI-based neuroimaging

Antibodies

Antibodies used	IL-2 mAb Alexa Fluor488 conjugate: Anti-IL2-Alexa488-ab (Invitrogen; RHCIL220); rabbit anti-Strep-tag IgG antibody: NWSHPQFEK Antibody, pAb, Rabbit, GenScript, A00626); Goat Anti-Rabbit-IgG-Alexa488: (Goat anti-Rabbit IgG (H+L) Cross-Adsorbed Secondary Antibody, Alexa Fluor™ 488, Invitrogen, A-11008); anti-Ubi-Fab-HRP: (AbyD03925; AbDSeroTec)
Validation	IL-2 mAb Alexa Fluor488 conjugate: Andersson, J., J. Abrams, L. Bjork, K. Funa, M. Litton, K. Agren, and U. Andersson. 1994. Immunology 83: 16. ; Fernandez, V., J. Andersson, U. Andersson, and M. Troye-Blomberg. 1994. Eur. J. Immunol. 24: 1808. NWSHPQFEK Antibody, pAb, Rabbit: Marion Avril, et al. Plasmodium falciparum adhesion domains linked to severe malaria differ in blockade of endothelial protein C receptor. Cell Microbiol. (2015-06)

Eukaryotic cell lines

Policy information about cell lines and Sex and Gender in Research

Cell line source(s)	Wi-38 (ATCC; CCL-75); NHDF (Promocell C-12302)
Authentication	for authentication procedure, see the responsible company
Mycoplasma contamination	all cell lines were tested negative for mycoplasma
Commonly misidentified lines (See ICLAC register)	*Name any commonly misidentified cell lines used in the study and provide a rationale for their use.*
